# An Evidence-Based Update on the Potential Association between Rheumatoid Arthritis and Lymphangioleiomyomatosis

**DOI:** 10.3390/jpm13040607

**Published:** 2023-03-30

**Authors:** Nicoleta Anamaria Pascalau, Andrei-Flavius Radu, Delia Carmen Nistor Cseppento, Felicia Liana Andronie-Cioara, Anamaria Jurcau, Calin Mos, Alexa Florina Bungau, Simona Gabriela Bungau

**Affiliations:** 1 Department of Psycho Neuroscience and Recovery, Faculty of Medicine and Pharmacy, University of Oradea, 410073 Oradea, Romania; nicoleta.pascalau@spitalpelican.ro (N.A.P.); felicia_cioara@yahoo.com (F.L.A.-C.); anamaria.jurcau@didactic.uoradea.ro (A.J.); 2 Doctoral School of Biomedical Sciences, University of Oradea, 410087 Oradea, Romaniasbungau@uoradea.ro (S.G.B.); 3 Department of Preclinical Disciplines, Faculty of Medicine and Pharmacy, University of Oradea, 410073 Oradea, Romania; 4 Department of Morphological Disciplines, Faculty of Medicine and Pharmacy, University of Oradea, 410073 Oradea, Romania; drmoscalin@yahoo.com; 5 Department of Pharmacy, Faculty of Medicine and Pharmacy, University of Oradea, 410028 Oradea, Romania

**Keywords:** rheumatoid arthritis, lymphangioleiomyomatosis, sirolimus, everolimus, pulmonary transplant, biological therapy

## Abstract

Lymphangioleiomyomatosis (LAM) represents an uncommon disorder characterized by cystic lung destruction and chronic respiratory failure. Lung damage caused by various mechanisms may represent a hypothesis for studying the association between LAM and rheumatoid arthritis (RA), which is the most prevalent autoinflammatory rheumatic disease and may affect the lungs as an extra-articular manifestation. Despite their distinct clinical presentations, the pathophysiology of both disorders includes dysregulated immunological function, abnormal cellular development, and inflammation. Current research suggests a potential relationship between RA and LAM, as some RA patients have been reported to develop LAM. However, the association of RA and LAM raises important therapeutic dilemmas. For this reason, the trajectory of a patient who was identified in our medical records as suffering from both LAM and RA, treated with many novel molecules and biological therapy, but with a negative outcome due to respiratory and multiorgan failure, has been exemplified. The delay in the diagnosis of LAM is due to a correlation between RA and LAM, worsening the vital prognosis and also hindering pulmonary transplantation. In addition, extensive research is essential for understanding the potential connection between these two disorders and discovering any similar mechanisms involved that may underlie their occurrence. This may contribute to the development of new therapeutic options that target shared pathways implicated in the pathogenesis of RA and LAM.

## 1. Introduction

The most frequent inflammatory rheumatic condition, rheumatoid arthritis (RA), affects about 1% of the entire population and is also one of the most-discussed topics among rheumatic diseases [1]. Furthermore, for women, the prevalence is 0.5 new diagnoses per 1000 people, while, for males, it is 0.2 new diagnoses per 1000 people, annually. RA damages the connective tissue leading to a symmetrical erosive synovitis of the joints and may also involve different systems and organs. The majority of subjects experience a chronic path of the illness, which alternates between times of aggravation and remission [2,3,4]. 

The overall management in RA, like other types of arthritis, can involve pharmacological treatment [5,6], rehabilitation treatment [7], surgical treatment [8,9], and numerous pharmacotherapeutic options offered by traditional therapies [10,11,12,13].

Left untreated, RA determines an evolving, irrevocable loss of joint function, persistent joint abnormalities, lower quality of life, and shorter life span. Moreover, visceral injuries generally reduce life expectancy by five to ten years [14,15].

The fact that over 50% of affected individuals cease working within the first five years of the onset of the disease and that 10% of individuals present serious incapacity within the first two years of progression is a significant negative effect of the illness. The diagnosis of RA needs to be promptly verified in accordance with The American College of Rheumatology (ACR)/European Alliance of Associations for Rheumatology (EULAR) classification standards, and therapy is required as rapidly as possible considering the possible gravity and negative outcomes [16].

Respiratory tract interference in RA is typically of the infiltrative variety, resulting in localized infiltrates or widespread pulmonary fibrosis and chronic obstructive disorders. Several other rheumatic disorders, such as systemic lupus erythematosus, mixed connective tissue disease (CTD), and Sjogren’s syndrome, also exhibit these symptoms. Furthermore, subpleural rheumatoid nodular destruction can result in pneumothorax. The pleural influence is typically clinically asymptomatic, but histological examinations frequently detect it at a preliminary phase, allowing for prompt treatment [16,17,18]. 

Interstitial lung injury occurs in 20–60% of individuals with RA, and periodic imaging scans demonstrate disease progression in 35–45% of patients. Pulmonary dysfunction in RA enhances patient death rates due to the paucity of therapies capable of reversing pulmonary injury, with the exception of biological therapies addressing cellular immune deficiency, which have limited effects [19]. In acute infections of the lower airways, a mixture of high-dose corticosteroids and appropriate antibiotics should be administered rapidly [20]. 

Lymphangioleiomyomatosis (LAM) is an uncommon condition with a strong female predominance, characterized by the noncancerous spread of atypical cells of smooth muscles across the thorax, along with the blood vasculature, pleura, lung parenchyma, and lymphatic vessels, resulting in damage to lung structure, cystic emphysema, and a gradual degradation of pulmonary function [21,22]. LAM occurs spontaneously, mainly between the ages of 20 and 40 [23], primarily among women of reproductive age [24].

Considering its clinical severity and the lack of targeted laboratory analyses, the occurrence of LAM is presumably underestimated. Nevertheless, the prognosis for LAM is poor. Current research suggests the life expectancy to be relatively close to 10 years, and spontaneous remission has even been reported [25,26]. However, according to information retrieved from the United Kingdom’s National Database, 10 years following the onset of LAM, 55% of patients had respiratory arrest of grade 3, and 10% had respiratory failure of grade 4. Moreover, 10 years after a lung biopsy, the rate of survival after 5 years is 85.1% [27]. 

Despite the fact that the pathophysiological mechanisms of this uncommon condition are poorly comprehended, multiple investigations have demonstrated a correlation between reproductive age and the beginning of LAM, indicating a potential estrogen pathway. Comparable to the process that leads to the proliferation of smooth muscle in the uterus throughout pregnancy, the hormone may be implicated in the aberrant development of muscle cells in the lungs [28,29,30]. 

Several potential mechanisms may explain the full implications of the association between RA and LAM, two pathologies without a fully acknowledged pathophysiological mechanism. RA and LAM are both linked to abnormal immune system activation [31,32]. In addition, chronic inflammation and oxidative stress may play a role in the development of both RA and LAM.

The purpose of the present research is to investigate the clinical implications of RA and LAM to update the data present in the scientific literature on possible correlations between the two pathologies. The choice of the research topic is based on the small number of publications treating LAM both as a rare disease and a possible correlation with RA, which also highlights the potential contributions to the current state of knowledge in the field. For exemplification, comprehension, and a better understanding of patient outcome, a comprehensive analysis of a rare medical case of a patient with RA and LAM is presented, highlighting the therapeutic possibilities in this rare association as well as the issues raised by the lack of consensus in therapeutic protocols and existing guideline recommendations for organ transplantation in autoimmune diseases.

## 2. Research Methodology and the Impact of Scientific Literature 

Considering the rarity of LAM, it is even more important to conduct related studies, use appropriate research methodologies, and have a comprehensive view of the currently available literature, as these aspects are crucial to advancing scientific knowledge about the disorder and enhancing patient outcomes.

The medical literature includes numerous evaluations of RA in different approaches, but it is deficient related to LAM and still insufficient if evaluating the literature addressing the association between these two pathologies.

The search algorithm is based on the following search terms: “autoimmune rheumatic diseases”, “rheumatoid arthritis”, “rheumatoid arthritis AND pulmonary manifestations”, “diagnostic, prognostic and theranostic of rheumatoid arthritis”, “pathophysiology of rheumatoid arthritis”, “therapeutic management of rheumatoid arthritis”, “rheumatoid arthritis AND DMARDs”, “lymphangioleiomyomatosis“, “lymphangioleiomyomatosis AND rheumatoid arthritis”, “lymphangioleiomyomatosis AND diagnosis”, “lymphangioleiomyomatosis AND pathogenesis”, “lymphangioleiomyomatosis AND clinical features”, “lymphangioleiomyomatosis AND therapeutic management”, and “lymphangioleiomyomatosis AND chronic inflammation”.

**Figure 1:**
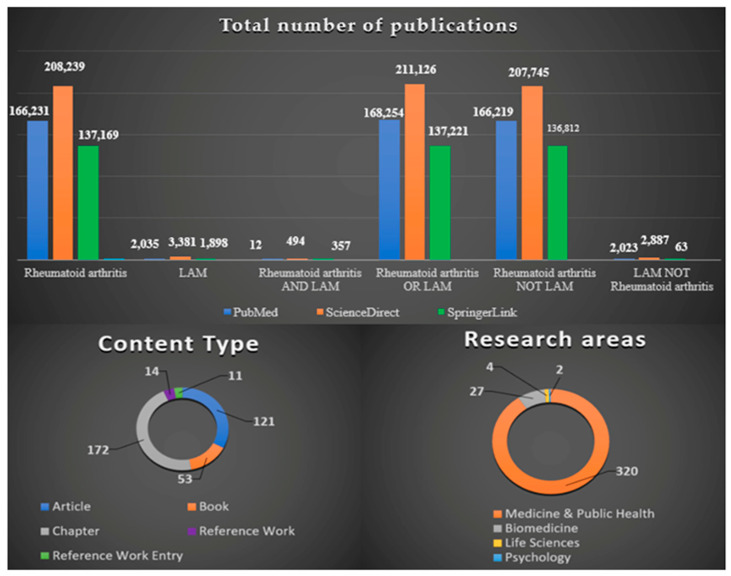
Literature impact on the RA-LAM association and relevant study areas and publication varieties highlighted via Boolean search tools in large databases. RA, rheumatoid arthritis; LAM, lymphangioleiomyomatosis.

A comparative analysis in terms of the total number of publications in three large and scientifically validated databases containing medical resources (i.e., PubMed, ScienceDirect, and SpringerLink) was performed based on the integration of the Boolean operators AND, OR, and NOT into the search algorithm. Furthermore, content type and research areas, according to SpringerLink, were introduced for the search “Rheumatoid arthritis AND Lymphangioleiomyomatosis” (Figure 1).

Overall, 94 bibliographic resources from the time interval 1984–2022 have been retrieved and acknowledged to support the output generated in this research.

The medical literature has emphasized the necessity for additional research to better comprehend the relationship between both disorders and to find efficient treatment options for people who have both RA and LAM.

## 3. Pathophysiological Mechanisms with Molecular Implications

### 3.1. Rheumatoid Arthritis

Even though the underlying etiology of RA is not completely determined, it is considered to result from a variety of hereditary, immunological, and environmental factors [33].

The pathophysiologic mechanisms of RA are characterized by an intricate interaction between multiple types of cells, mediators, chemokines, signaling pathways, and cytokines, which results in inflammatory processes, bone and cartilage damage, and also systemic implications leading to extra-articular manifestations [34].

Various cells with immunological functions (i.e., B cells, macrophages, and T cells) [35] infiltrate the synovial membrane and discharge pro-inflammatory cytokines, such as tumor necrosis factor-alpha (TNF-α) and both interleukin (IL) 1 and 6, triggering one chain reaction of occurrences that enhance the inflammatory process and promote pathological changes [36]. Furthermore, chemokines, including chemokine (C-C motif) ligand 2, IL-8, and chemokine (C-C motif) ligand 12, have an essential function in the recruitment and retention of immune cells in the synovium [37].

The osteoprotegerin pathway, which controls osteoclast development and bone resorption, and the receptor activator of nuclear factor κB ligand are additional factors that are essential in the pathophysiology of RA. In RA, there is a disproportion involving receptor activator of nuclear factor κΒ, which is highly expressed by immune cells and fibroblasts, and its evasion receptor, osteoprotegerin, which causes excessive bone loss and degradation [3,38].

Several susceptibility genes, including protein tyrosine phosphatase non-receptor type 22 [39], tumor necrosis factor alpha-induced protein 3 [40], and human leukocyte class II histocompatibility antigen, DRB1 beta chain [41], have been identified as the genetic basis of RA. It is therefore considered that these genetic variations influence immunological activity, antigen presentation, and mediator signaling, hence raising the possibility of developing RA and regulating disease severity.

The development of RA is an intricate and evolving process involving different variables and mechanisms. Understanding these pathways is essential for the development of personalized medicines that can successfully reduce inflammation, maintain joint mobility, and improve RA patients’ quality of life.

### 3.2. Lymphangioleiomyomatosis

LAM cells proliferate in the pulmonary and lymphatic systems, leading to the degradation of lung tissue, poor gas exchange, and respiratory distress. The precise molecular mechanism leading to LAM is not completely understood, although it is considered to be triggered by variations within the tuberous sclerosis complex (TSC1/TSC2) genes, which influence the stimulation of the mammalian target of rapamycin (mTOR) signal transduction pathway [42,43].

The pathophysiology of LAM is described by a complex interaction between LAM cells, inflammatory cytokines, growth regulators, and signal transduction pathways, which results in the abnormal proliferation, movement, and infiltration of LAM cells, as well as the restructuring of lung tissue. LAM is characterized by the invasion of lung tissue by LAM cells, which display smooth muscle indicators, such as desmin and alpha-smooth muscle actin, and release proliferation promoters, such as vascular endothelial growth factor-D (VEGF-D) and insulin-like growth factor-1. The above-mentioned growth factors induce lymphangiogenesis and tissue restructuring, hence facilitating the development of LAM characteristics such as cystic lesions, lymphatic constriction, and bronchiolar wall thickening [44,45,46]. 

The mTOR signaling cascade, which is poorly controlled in LAM cells because of changes in TSC1/TSC2, activates downstream effectors, including 4E-binding protein 1 and ribosomal protein S6 kinase beta-1. The mTOR pathway regulates cellular proliferation and survival, and its subsequent activation in LAM cells plays an important role in their abnormal development, mobility, and invasion, as well as the interference of the extracellular matrix and the initiation of pro-inflammatory cytokine responses [47]. 

Estrogens may have a significant role in the pathophysiology of LAM, given that LAM develops mainly in women. In addition, it has been demonstrated that LAM pulmonary nodules and angiomyolipoma possess progesterone and estrogen receptors. In addition, LAM is commonly found in premenopausal women, and pulmonary symptoms have been shown to intensify during pregnancy or after the injection of exogenous estrogens [48,49].

The pathophysiology of LAM is a complex process targeting multiple factors and pathways, and acknowledging the mechanisms underlying its development is essential for improving its overall management.

## 4. Diagnosis and Pharmacotherapeutic Management

### 4.1. Rheumatoid Arthritis

RA is associated with ongoing joint inflammation, discomfort, and rigidity, which can result in joint injury, deformities, and disability. Clinical examination, laboratory investigations, and imaging techniques are used to diagnose RA [50]. 

The physical examination commonly consists of assessing joint swelling, pain, and flexibility. Moreover, inflammatory biomarkers, including erythrocyte sedimentation rate (ESR) and C-reactive protein (CRP), and particular Y-shaped antibodies, such as anticyclic citrullinated peptide antibodies (ACPA) and rheumatoid factor (RF), can also be assessed in the blood samples. Magnetic resonance imaging, ultrasounds, and X-ray examinations can be used for identifying joint injuries and inflammatory conditions [51,52,53]. 

Current improvements in the pharmacological and pharmacotherapeutic treatment of RA are derived from continual research into drug development technologies, with pain control, slowed disease progression, and remission of pathology as the ultimate outcomes. The most beneficial pharmacological treatments for RA include symptomatic treatment with nonsteroidal anti-inflammatory medications (i.e., ibuprofen, celecoxib, etc.) and glucocorticoids (i.e., dexamethasone, prednisone, etc.), as well as disease-modifying antirheumatic drugs (DMARDs), which can be synthetic (i.e., methotrexate, leflunomide, etc.), targeted synthetic (i.e., tofacitinib, upadacitinib, baricitinib, etc.), or biological (i.e., infliximab, etanercept, tocilizumab, rituximab, etc.) [1,3].

Although there is no fully effective RA treatment, pharmacotherapeutic interventions may alleviate symptoms and slow progression.

### 4.2. Lymphangioleiomyomatosis

Due to its infrequent occurrence and nonspecific symptoms, LAM can be difficult to diagnose. Blood tests, pulmonary function tests, radiological evaluations, and biopsies can be performed to confirm the diagnosis of LAM [32,54].

Laboratory assessments should involve tests to exclude several circumstances, such as ACPA and RF, which can be related to lymphoid interstitial pneumonia, and α1-antitrypsin depletion, which is correlated with emphysema [32]. Moreover, the lymphangiogenic VEGF-D was discovered to be increased in LAM [54].

Respiratory function measurements are essential for estimating the extent of lung damage and guiding therapies [55].

Thoracic X-rays are frequently normal or may reveal a small rise in interstitial biomarkers or pleural effusion in the initial phases of the illness. In a chest computed tomography, thin-walled cysts are critical for diagnosing pulmonary LAM [56].

Lung or lymph node tissue can be obtained in biopsies to study the cells and confirm the diagnosis of LAM [24].

The American Thoracic Society and the Japanese Respiratory Society published clinical practice guidelines in 2016 stating that a correct diagnosis of LAM can be made if the patient has a consistent case history: youthful to middle-aged, female, with exacerbating dyspnea and/or pneumothorax or chylothorax despite the apparent lack of characteristics evocative of other cystic respiratory ailments and has a characteristic high-resolution computed tomography image of the chest. Moreover, it is essential that one of the following can be identified: lymphatic malformations, abdominal or thoracic chylous effusions, renal angiomyolipoma, tuberous sclerosis, proof of LAM cells or LAM cell clusters on cytological analysis of effusions or the lymphatic system, or histological evidence of LAM by lung biopsies or biopsy of retroperitoneal or pelvic masses [57]. When an increasing lot of individuals are examined using international LAM information registries, the standard image of this condition tends to be evolving. A greater understanding of LAM and its typical clinical manifestations may facilitate the design of advanced treatment approaches and decrease the number of patients who are incorrectly diagnosed [26]. Considering the afore-mentioned diagnostic criteria, the estimated prevalence of LAM within Japanese patients is between 1.2 and 2.5 per million [57].

Based on the observation of the key role of increased mammalian target of rapamycin complex 1 (mTORC1) signaling in LAM pathophysiology, sirolimus (mTORC1 inhibitor) is being evaluated as a therapeutic option for LAM. Furthermore, a 1-year treatment of LAM with sirolimus decreased the angiomyolipoma progression by approximately 50%, according to a pilot study [58,59].

Rapamune^®^ is the trade name for the drug, which contains the active substance sirolimus and was approved as an orphan drug on July 14, 2016 (EU/3/16/1704) against sporadic LAM [60]. Sirolimus reduces T-cell activation caused by the majority of stimuli by inhibiting calcium-independent and calcium-dependent intracellular signaling pathways. According to medical data, its effects are controlled by mechanisms distinct from those observed with tacrolimus or cyclosporine [61]. 

Research suggests that sirolimus attaches to the cytosolic-specific molecule FKPB-12. Furthermore, the FKPB-12 sirolimus structure blocks the stimulation of mTOR, which is an essential protein for the function of the cell cycle. Suppression of mTOR affects particular signaling pathways, which reduces lymphocyte stimulation and results in immunosuppressive effects [62]. A multicenter clinical trial showed the effectiveness of sirolimus in maintaining pulmonary function, enhancing the quality of life, and decreasing lymphatic symptoms in LAM patients [63]. 

In this context, new recommendations made by the Japanese Respiratory Society and American Thoracic Society for the therapy of individuals with LAM and compromised respiratory function call for sirolimus [15]. Yet, there are significant restraints on the long-term use of sirolimus for patients, due to worries about safety and tolerance [64]. 

To identify the optimal sirolimus dosage regimen for LAM patients that maximizes effectiveness and optimizes the potential for side effects, additional research is required [65].

Everolimus is an alternative to sirolimus, with a similar mechanism of action. They both stabilize pulmonary function and decrease the prevalence of renal LAM. They both reduce the incidence of renal LAM and regulate pulmonary function. Subjects taking sirolimus or everolimus could have a mean transplant-free life expectancy of around 29 years following the onset of the illness and a mean 10-year transplant-free survivability of 86% [66]. It is still unknown whether the combined regimen (everolimus/sirolimus with triptorelin) is safe and effective [67].

Around 20% of LAM patients showed reversible air flow obstruction, and a treatment trial with bronchodilators must therefore be evaluated for symptomatic patients. Moreover, based on medical information acquired from a case report, it was considered that doxycycline was a promising treatment for LAM due to its activity against multiple matrix metalloproteinases (MMPs) [68,69]. 

Preliminary research on statins and cyclooxygenase-2 selective inhibitors were reported, yet additional, extensive investigations are required to assess the efficacy and safety profiles [70,71].

However, pulmonary transplant (PT) is still a viable choice for patients with advanced LAM, greatly enhancing their standard of living [72]. PT has been increasingly used over the past 20 years in subjects in which autoimmune CTDs determined pulmonary fibrosis or serious lung tissue injury. Pulmonary damage is frequent in CTDs, and respiratory failure is a leading contributing factor to mortality and morbidity in CTD-related interstitial lung disease (CTD-ILD) [73]. A study performed in Korea found similar survival rates for patients undergoing pulmonary transplant either for idiopathic pulmonary fibrosis or CTD-ILD [74]. Other studies have reported superior results in PT for LAM compared to other indications [75,76,77].

In the context of evaluating the management of RA and LAM, information obtained from medical cases managed by the present authors in which patients with both pathologies were evaluated becomes essential in investigating possible correlations between apparently unrelated conditions.

## 5. Evidence-Based Assessment of a Patient with RA and LAM

This study assessed a 30-year-old nonsmoking female who presented to the Rheumatology Ward complaining of joint pain in both hands (II/III metacarpophalangeal joints, II/III/IV interphalangeal joints), muscular and knee pain, stiffness of her joints for over 90 min each morning, severe tiredness, and decreased force of finger grip. Being pregnant, her symptoms began with the first trimester, two months ago. No significant family or past medical history was detected. 

**Figure 2:**
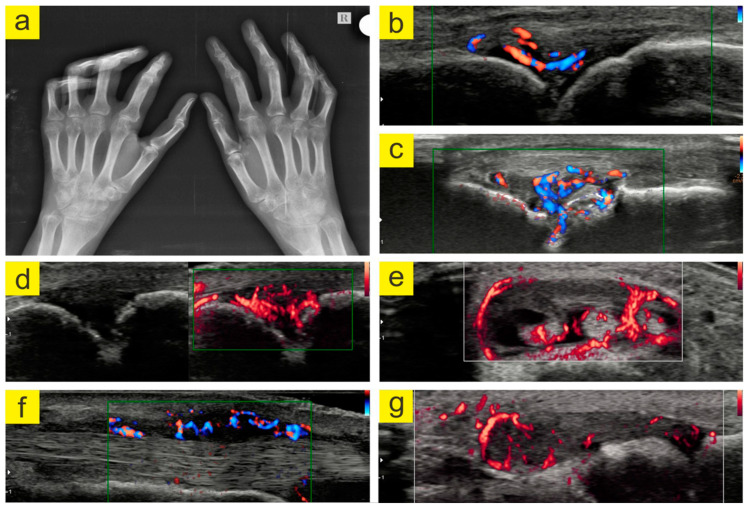
(**a**). Bilateral AP hand radiography. Radiological imaging identified multiple marginal erosions; narrowing of the proximal and distal interphalangeal, metacarpophalangeal, carpo-metacarpal, and carpal bone joints in both hands; osteoporosis in the band; subluxation proximal interphalangeal II, III, IV, and V of the left hand; proximal interphalangeal IV and V of the right hand, and metacarpophalangeal IV and V of the right hand. (**b**). Hyper vascular synovial pannus (directional power Doppler). (**c**). Synovial pannus and bone erosion (directional power Doppler). (**d**). Hyper vascular synovial pannus (microvascular flow). (**e**). Tenosynovitis extensor tendons of the fingers (microvascular flow). (**f**). Synovial pannus radial extensor of the carpus and ulnar extensor of the carpus. (**g**). Synovial pannus at the MTF I joint level (microvascular flow). (Images archive of the first author).

On general clinical examination, everything related to blood pressure, temperature, the superficial ganglion system, integuments and mucous membranes, subcutaneous adipose tissue, the circulatory system, respiratory system, gastrointestinal system, genital and urinary systems, and nervous system was within normal limits. Laboratory studies identified a significant inflammatory syndrome with a more than 3-fold elevation of the CRP levels (25 mg/L) and ESR (45 mm/h), while the levels of RF (45 IU) and anti-ACPA (40 U/mL) were increased more than 5-fold. Hematological parameters (i.e., leukocytes, red blood cells, platelets) were within normal limits. In both hands, at the level of the small joints, inflammation and active synovitis were discovered by musculoskeletal ultrasonography (Figure 2).

Taking into account the RA classification criteria mentioned in ACR/EULAR 2010, initially a diagnosis of RA resulted [28], but, being in the third month of pregnancy, the subject rejected DMARDs and anti-inflammatory treatment initiation. 

After 10 months, the patient returned with an altered functional status, evaluated according to all three protocols (Disease Activity Score (DAS 28), Health Assessment Questionnaire, and Visual Analogue Scale (VAS)), reporting morning joint stiffness to last over 120 min. The women’s clinical investigation indicated numerous swollen and sore joints, the presence of rheumatoid nodules in the elbow, and severe functional impotence in the hands and feet, as presented in Figure 2. The laboratory studies showed further increases in the levels of RF (140 IU) and ACPA 100 U/mL, both of which were more than 10-fold increased, a more than 5-fold increase in the CRP levels (26 mg/L), an ESR value of 80 mm/h, and a 25 OH vitamin D value of 26 ng/mL. 

Weekly treatment with 20 mg methotrexate (MTX) + 5 mg folic acid and corticosteroids (i.e., prednisone) (0.5 mg/kg body weight) was initiated, along with vitamin D at 1000 IU/day, with a slight improvement of the joint pain and swelling. It is worth noting that in patients with RA vitamin D supplementation is advised, but there is an ongoing debate as to whether serum 25(OH) vitamin D levels correlate with disease severity [78]. 

**Figure 3:**
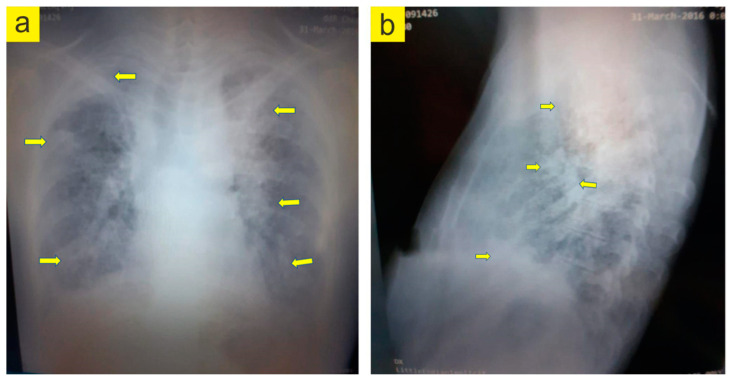
Chest radiography. (**a**). PA lung grapy. (**b**). Lateral lung grapy. Yellow arrows indicate the problem areas. (Images archive of the first author).

**Figure 4:**
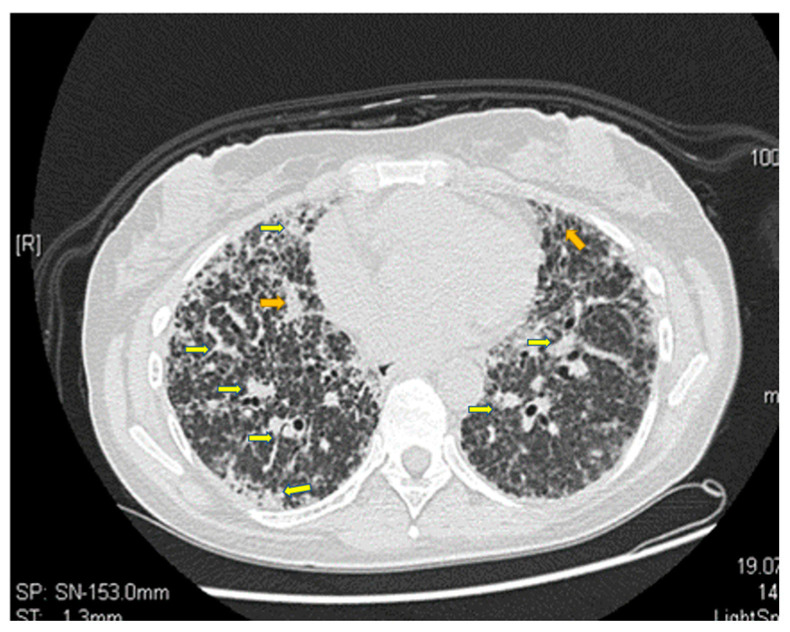
Infrahilar axial section. Polymorphous bilateral pulmonary appearance with marked augmentation with thickening of irregularly shaped interstitial septa and local pulmonary tractions. Yellow arrows indicate the problem areas. (Computed tomography scan of the lungs from the archive of the first author).

The patient reported experiencing loss of appetite, vomiting, malaise, and nausea, following the MTX intake, at the three-month follow-up. The results of the clinical exam were DAS 28: 5.9, SJC: 6, VAS: 70, and TJC: 4, where SJC represents the number of swollen joints and TJC is the number of sensitive joints. In addition to the side effects of MTX, the patient complains of dyspnea on exertion. Laboratory studies showed a persistent inflammatory syndrome, with an RF of 356 IU and ACPA levels of 1245 IU/mL (within normal ranges), and a mild normochromic normocytic anemia. Chest radiographies (Figure 3 and Figure 4) showed both widespread fibrosis associated with a “honeycombing” aspect at the pulmonary level and many thin-walled cysts.

Due to the side effects, MTX was discontinued and leflunomide was initiated at 20 mg/day instead in association with sulfasalazine at 3 g/day, corticosteroids (i.e., prednisone at 10 mg/day), and bronchodilators by nebulization (i.e., ventolin-salbutamol, 2 × 100 μg of salbutamol, 4 times a day).

After another 3 months, the patient returned to check-up, still complaining of joint pain and swelling (i.e., hands and feet), persistent dyspnea, severe fatigue, and functional impotence. In addition, she reported hair loss and hemoptysis. Given the lack of response to two DMARDs in maximum doses over 3 months, the patient was screened for pulmonary tuberculosis and both hepatitis B and C, which were all negative; adalimumab, a TNF-α- targeted monoclonal antibody, was started at 40 mg/2 weeks in combination with leflunomide at 20 mg/day, nonsteroidal anti-inflammatory medicines (i.e., naproxen at 1100 mg/day), and inhaled bronchodilators (i.e., salbutamol at 800 μg/day). 

Three months later, the joint inflammatory syndrome improved clinically/biologically and the joint discomfort and swelling partially subsided (the following results were obtained: CRP: 6.8 mg/L, DAS 28: 2.1, ESR: 29 mm/h, VAS: 22, SJC: 2, TJC: 3), but frequent infections at the respiratory level and severe dyspnea occurred, requiring antibiotics (i.e., levofloxacin, 750 mg IV daily for 10 days, 3 episodes of infection in 3 months) and salbutamol nebulizer treatment. 

Evaluation of the respiratory system with functional explorations and CT scan revealed a progressive deterioration of the respiratory function (Figure 4). The parameters evaluated by spirometry show FEV1 (the maximum volume expired in the first second) < 55%, forced vital capacity (FVC) of 60%, PEF (maximum flow obtained after a maximum forced expiration - expression of air flow in the small airways) of 50% and a requirement for intermittent oxygen therapy. 

Given the increased risk of infection associated with adalimumab and leflunomide, especially in patients with altered respiratory function, these 2 drugs have been discontinued and the treatment prescribed was as follows: cyclophosphamide at 200 mg/day in association with hydroxychloroquine at 400 mg/day, methylprednisolone at 16 mg/day, and colchicine at 1 mg/day.

However, the condition of the patient continued to deteriorate, and the subject presented with repeated episodes of hemorrhagic cystitis as a side effect of cyclophosphamide treatment, lost around 5 kg in 4 weeks, and continued to complain of hair loss, while her hand, knee, and elbow joints ached and were swollen (ESR: 85 mm/h, DAS 28: 6.1, SJC: 14, TJC: 12). Under these circumstances, the medical team decided to start a therapeutical approach using rituximab (chimeric monoclonal antibody), plus hydroxychloroquine, the combination of the two drugs being advised in the cases of adult patients’ treatment for severe/active RA and an intolerance or inappropriate response to other DMARDs or TNF-targeted drugs. 

**Figure 5:**
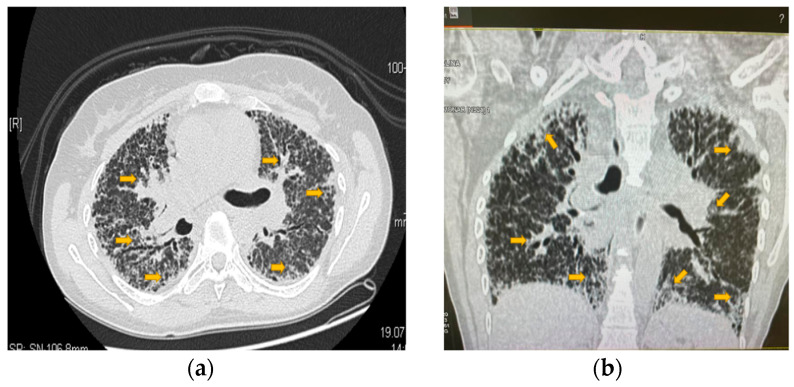
Highly accentuated bilaterally symmetric reticulo-micronodular peri broncho vascular interstitium with marked thickening of the interlobular septa and ground-glass images in the context of pulmonary lymphangitis and extensive pulmonary fibrosis changes. (**a**): Section in the floor of the pulmonary hilum; (**b**): reconstruction through the floor of the pulmonary hilum. Yellow arrows indicate the problem areas. (Computed tomography scan of the lungs from the archive of the first author).

Our patient received 1000 mg delivered in an intravenous infusion, repeated after 2 weeks, being reassessed after one month with laboratory studies and chest computed tomography scan (Figure 5). The scan showed pulmonary fibrosis with a “matte glass” appearance, which is why the lung biopsy was performed. In order to allow the exclusion of associated micro-thrombotic processes, ESR and CRP values were markedly elevated, while antinuclear antibodies, human leukocyte antigen B27 (a myositis profile for excluding antisynthetase/myositis syndrome), perinuclear antineutrophil cytoplasmic and antineutrophil cytoplasmic antibodies, and anticardiolipin antibodies, were all negative. Moreover, VEGF-D was 800 pg/mL. 

**Figure 6:**
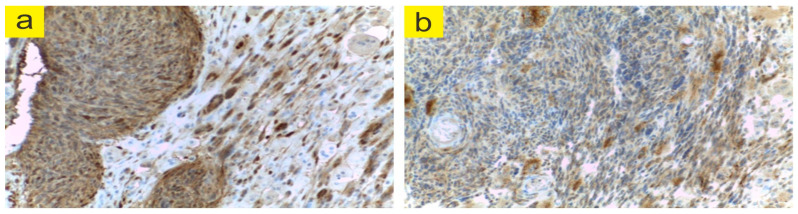
Histopathological modifications in a patient with LAM and RA (images archive of the first author). SMA positive (**a**). on the described nodular areas; CD34 positive (**b**). on the wallpaper of the collapsed cystic spaces. (Images archive of the first author).

The examined material (Figure 6) is represented by two fragments of bronchial mucosa, one of which shows epithelial erosions and stromal edema with little chronic inflammatory infiltrate. The second fragment shows a fibrous stroma containing probably collapsed cystic spaces, some of which have fasciculate nodules with an eosinophilic appearance in the walls. The histological appearance correlated with the immunohistochemical profile may belong to a LAM.

After these investigations, the clear conclusion was that the respiratory function’s deterioration was not because of RA, but because of LAM. As a result, monitoring of the patient’s blood levels was performed as the sirolimus (2 mg/day) was initiated [32]. Unfortunately, the patient’s general condition continued to deteriorate, showing additional swellings and joint soreness (SJC: 10, TJC: 14); her pulmonary function kept getting worse as well. The patient’s assessment by spirometry revealed FEV1 < 35%, FVC of 60%, and PEF of 30% and she required continuous oxygen therapy. Moderate pulmonary hypertension and numerous insufficiencies (acute respiratory, 1^st^ degree mitral and 1^st^ degree tricuspid) continued to occur. ESR (110 mm/h) and CRP (15.88 mg/L) were markedly increased. Having severe oxygen-dependent respiratory failure, the subject was considered to be in need of a lung transplant. The patient died while awaiting a lung transplant, after 10 months from the respiratory symptoms’ onset and 3 years from the RA onset.

The patient’s clinical evaluation is briefly depicted in Table 1, while Table 2 provides the dynamics of the biological disease markers and the repeated changes of therapy. 

Taking into account the increased values of reactants (in the acute phase) and levels of RF and ACPA antibodies, the presence of bone erosion on the radiographic evaluation, and the age of onset, a potential unfavorable course could have been expected. Initially, the respiratory symptoms have been attributed to the systemic involvement of RA and the treatment protocol adapted by discontinuing MTX and initiating biological treatment with cortisone and adalimumab [6]. The unfavorable respiratory evolution (i.e., frequent respiratory infections, fever, hypoxia/respiratory distress, weight loss over a few months, and fever) and favorable joint evolution raised the suspicion of another cause of pulmonary distress, leading to repeated imagistic evaluation and lung biopsy. However, despite histological confirmation of LAM and the initiation of treatment with sirolimus, the clinical status of the patient continued to deteriorate and had an unfavorable outcome 3 years after RA diagnosis and 10 months after starting treatment for LAM. Moreover, the evolution of LAM was fulminant, leading to pulmonary tissue destruction and severe respiratory failure, followed by multiorgan failure and death, and lung tissue destruction was fulminant. Severe pulmonary damage and oxygen-dependent respiratory failure led to multiorgan failure and death. PT, which might have saved the patient’s life, was not available in due time. 

In Romania, few patients with severe respiratory diseases can access PT. According to the National Transplant Agency, only four procedures were performed in 2018 and three in 2019, two in 2020, and two in 2021. Currently, five patients are waiting for a lung transplant from a compatible donor [79].

Currently, there are no absolute guidelines, the selection process may vary by center, and the waiting list, the procedures of the center, and other variables all playing an essential part in making the decision to perform an organ transplant. Understanding the intricate molecular processes underlying autoimmune conditions has been the subject of extensive study, yet, to determine the involvement of pathogenic pathways associated with mitochondrial function and oxidative stress [80] in the pathogenesis of autoimmune disorders and their relationship to the chronic inflammatory condition, additional research is required [81]. As these molecular mechanisms are acknowledged and thoroughly comprehended, new approaches for attempting to enhance the life expectancy of these patients will be unequivocally revealed.

## 6. Common Features of Rheumatoid Arthritis and Lymphangioleiomyomatosis 

Although LAM is not confirmed to be causally triggered by RA, there have been several studies suggesting a link between the two disorders [3,82,83].

According to a clinical study, RA patients had a greater frequency of LAM compared to the overall population. Out of 68 patients with RA, 4, whose medical records were examined for several studies, had a confirmed diagnosis of LAM, translating to a prevalence rate of 5.9% versus the expected prevalence rate of 0.3–3.3% in the wider population [24,84].

The medical literature has contributed to raising awareness of the relationship between RA and LAM and the importance of LAM screening in RA patients. Several patients’ LAM diagnoses were made early as a result, and their pulmonary conditions were better managed [54,85]. Moreover, the scientific literature includes publications in which the safety and efficacy profiles of drugs for RA patients have been examined [3], but evaluations have also been conducted for LAM therapy [66], providing essential information that may be combined in order to optimize the management of patients who have both RA and LAM at the same time. Additionally, published data helped in the determination of potential comorbidities. Pulmonary hypertension and interstitial lung disease have been noted in the literature as potential comorbidities of LAM in patients with RA [25,63,85].

A single report of LAM being associated with RA was identified out of 152 cases of RA in a follow-up investigation of a group receiving medical care at the National Hospital Organization Kinki-Chuo Chest Medical Center. All subjects with LAM were female [83]. There was no estimate of the occurrence or frequency of LAM in the overall community in any of the mentioned studies.

In-depth evaluation of different molecules with pathophysiological implications identified a few similar processes in RA and LAM.

VEGF is a significant growth factor specific to endothelial cells that is increased by inflammatory mediators and hypoxia. In RA, serum VEGF levels are increased and correspond with disease activity [86]. Moreover, the clinical practice guidelines of the Japanese Respiratory Society and the American Thoracic Society promote, when LAM is considered, the use of blood VEGF-D as a diagnostic instrument, regardless of the absence of additional reliability and validity characteristics such as angiomyolipoma and lymphangioleiomyoma [87].

RA is three times more prevalent in women than in men, and a significant correlation to hormone levels has been established. There is substantial evidence that autoimmune processes are under genetic regulation, and genes on sexual chromosomes may contribute to the female predominance. In addition, it is generally acknowledged that estrogens may influence the immune reaction by supporting the survival of restricted autoreactive clones and eventually the preponderance of autoimmunity in women [88]. Furthermore, the female sexual dimorphism of LAM in conjunction with both the period of onset (women of reproductive age) and the identification of the expression of estrogen and progesterone receptors in LAM cells indicates that estrogen signaling is essential to the growth and evolution of LAM [89].

Enhanced osteoclast development and activity cause bone degradation. Fibroblast-like synoviocytes (FLSs) are specialized cells that can generate MMP once triggered. As a feedback control, MMP can cause cartilage deterioration, and cartilage also secretes proteases. FLSs may spread from one joint to another, resulting in a symmetrical distribution of RA [90]. In addition, it is considered that proteolytic enzymes generated by LAM cells play a crucial role in the production of pulmonary parenchymal cysts. Current research suggests that MMPs may be partially responsible for this harm [91].

When RA patients were assessed in comparison with healthy individuals, their CD8+ cell p-mTOR levels were higher, and those levels were positively correlated with the gravity of their condition. These results suggest that CD8+-cell-based mTOR activation could represent a prospective biological marker for RA condition severity assessment and an indicator of TNF inhibitors’ treatment response [92].

The invading cell in LAM has genetic variations in TSC genes that occur in the upregulation of the mTOR pathway and dysregulated cellular proliferation, leading to dysfunctional lung remodeling and respiratory arrest [32].

**Figure 7:**
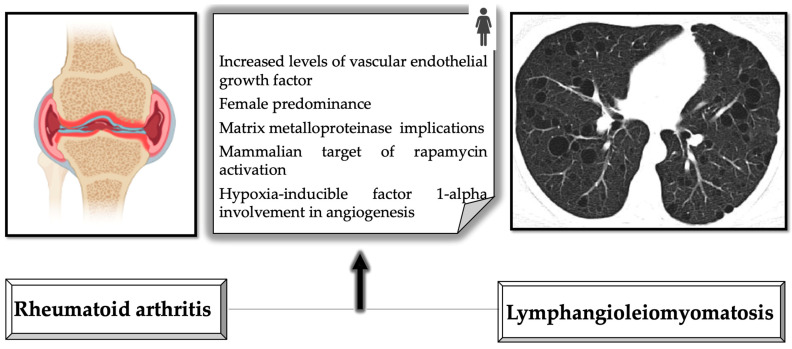
Description of shared processes in RA and LAM.

High-mobility group box protein 1 (HMGB1) has been investigated for its novel potential role in modulating hypoxia-inducible factor (HIF)-1 in facilitating angiogenesis in RA synovium. Administration of an anti-HMGB1 neutralizing antibody reduced collagen-induced arthritic symptoms in the impacted joint regions of mice models and inhibited the development of blood vessels in conjunction with a decrease in HIF-1α expression. The findings support the hypothesis that an elevation in HMGB1 triggers a rise in inflammatory synovium by enhancing HIF-1α activation and angiogenesis in RA. Therefore, blocking HMGB1 may be beneficial for the treatment of RA-related angiogenesis [93]. Moreover, in LAM assessments, mTOR, ribosomal protein S6 kinase beta-1, and eukaryotic translation initiation factor 4E-binding protein 1 are phosphorylated and activated because of the formation of mTORC1. These events boost the expression of HIF-1α proteases [94]. Figure 7 depicts shared processes of RA and LAM.

There is increasing evidence of similarities between the molecules implicated in the pathophysiology of RA and LAM, suggesting a potential association between both disorders that requires further study.

## 7. Conclusions

Although RA and LAM are two different disorders with various clinical symptoms, they show similarities in their pathophysiology, notably in respect to dysregulated immune system function, abnormal cellular development, and inflammatory processes.

The exemplification case study provides an in-depth analysis of a rare occurrence of concurrent RA and LAM through analyzing the patient’s medical records, imaging evidence, laboratory test results, and clinical outcomes. Therefore, it may contribute to a better understanding of the potential link between these two disorders and would promote the evaluation of potential risk factors, clinical characteristics, and treatment techniques that may be effective in managing these patients. Researchers should not dismiss the possibility that an earlier diagnosis of LAM could have provided individuals with the opportunity to undergo a lung transplant.

Numerous future research approaches may be investigated to advance the current understanding of the relationship between RA and LAM (i.e., large population-based analyses for the provision of more robust estimates, mechanistic studies to investigate molecular processes and potential targets, the standardization of screening and diagnostic guidelines, long-term outcomes, etc.). Throughout the recognition of the suspected association between RA and LAM, healthcare professionals should therefore be cautious for the co-occurrence of these disorders and acknowledge screening RA patients for LAM, specifically if respiratory symptoms or a loss in pulmonary function are observed. This aspect also highlights the necessity for a multidisciplinary approach to the management of patients with these disorders.

## Figures and Tables

**Table 1:** The patient’s clinical evaluation during the disease course.

Scheme 28.	Painful/Swollen Joints (Number)	VAS	DAS 28
Inflammation and symmetrical joint pain, bilateral functional hand deficit and stiffness over 90 min, and neurasthenia (I and II)	12/12	70	5.57
	11/15	90	5.1
Loss of appetite, vomiting, nausea, dyspnea on exertion, and discomfort after methotrexate administration (III)	6/9	70	5.9
Decreased muscle strength, persistent dyspnea, hair loss, hemoptysis, and severe neurasthenia and/or depression (IV)	10/15	90	5.88
Respiratory infections and severe dyspnea (V)	3/2	20	2.1
Weight loss (approx. 5 kg/4 weeks); hair loss; both hands, elbows, and knees present polyarthralgia + swelling of the small joints; cyclophosphamide treatment followed by frequent hemorrhagic cystitis (VI)	14/12	80	6.1

DAS 28, Disease Activity Score; VAS, Visual Analogue Scale.

**Table 2:** Summary of the patient’s biological markers of disease in dynamics and the changes in the treatment protocol.

ESR mm/h/ CRP mg/L	RF UI	ACPA U/mL/VEGF-D pg/mL	Treatment (Visit Number)
45/25	70	40/Nd	Refused (I)
80/26	140	100/Nd	Methotrexate at 20 mg/week, corticosteroids (Prednison) at 0.5 mg/kg body weight, folic acid at 5mg/week, and vitamin D at 1000 IU/day (II)
80/25	356	1245/Nd	Daily Leflunomide at 20 mg + Prednison at 10 mg + Sulfasalazine at 3 g and Salbutamol at 400 µg (nebulizer treatment) (III)
75/30	Nd		Adalimumab at 40 mg (1 ampoule)/2 weeks, daily Leflunomide at 20 mg + Naproxen 1100 mg, and Salbutamol at 800 µg (nebulizer treatment) (IV)
29/6.8			Daily Cyclophosphamide at 200 mg + Hydroxychloroquine 2 × 200 mg + Medrol at 16 mg + Colchicine at 1 mg (V)
85/20			Rituximab at 1000 mg, i.v., repeated after 2 weeks and Hydroxychloroquine at 400 mg/day (VI)
90/30	Nd	Nd/≥800	Daily Hydroxychloroquine at 400 mg + Sirolimus at 2 mg (VII)

ACPA, anticitrullinated protein antibodies; CRP, C reactive protein; ESR, erythrocyte sedimentation rate; Nd, not determined; RF, rheumatoid factor; VEGF-D, vascular endothelial growth factor D; i.v., intravenous.

## Data Availability

Data presented can be found in the archive of the Pelican Hospital Oradea, Romania, images can be found in the archive of the first author, and the literature information can be found in the inserted references.

## References

[1] Radu A.F., Bungau S.G., Negru P.A., Marcu M.F., Andronie-Cioara F.L. (2022). In-depth bibliometric analysis and current scientific mapping research in the context of rheumatoid arthritis pharmacotherapy. Biomed. Pharmacother..

[2] Guo Q., Wang Y., Xu D., Nossent J., Pavlos N.J., Xu J. (2018). Rheumatoid arthritis: Pathological mechanisms and modern pharmacologic therapies. Bone Res..

[3] Radu A.F., Bungau S.G. (2021). Management of Rheumatoid Arthritis: An Overview. Cells.

[4] Kvien T.K., Uhlig T., Ødegård S., Heiberg M.S. (2006). Epidemiological aspects of rheumatoid arthritis: The sex ratio. Ann. N. Y. Acad. Sci..

[5] Hua C., Buttgereit F., Combe B. (2020). Glucocorticoids in rheumatoid arthritis: Current status and future studies. RMD Open.

[6] Teaha D.I.M., Pascalău N.A., Lazăr L. (2019). Comparative study of the clinical response of patients to different treatment regimens in rheumatoid arthritis. Farmacia.

[7] Hammond A. (2004). Rehabilitation in rheumatoid arthritis: A critical review. Musculoskeletal Care.

[8] Leon L., Abasolo L., Carmona L., Rodriguez-Rodriguez L., Lamas J.R., Hernandez-Garcia C., Jover J.A., Alegre J., Alonso J.L., Alvarez M. (2013). Orthopedic surgery in rheumatoid arthritis in the era of biologic therapy. J. Rheumatol..

[9] Trieb K., Hofstaetter S.G. (2009). Treatment strategies in surgery for rheumatoid arthritis. Eur. J. Radiol..

[10] Bungau S.G., Behl T., Singh A., Sehgal A., Singh S., Chigurupati S., Vijayabalan S., Das S., Palanimuthu V.R. (2021). Targeting Probiotics in Rheumatoid Arthritis. Nutrients.

[11] Sharma T., Sharma P., Chandel P., Singh S., Sharma N., Naved T., Bhatia S., Al-Harrasi A., Bungau S., Behl T. (2022). Circumstantial Insights into the Potential of Traditional Chinese Medicinal Plants as a Therapeutic Approach in Rheumatoid Arthritis. Curr. Pharm. Des..

[12] Wang Y., Chen S., Du K., Liang C., Wang S., Owusu Boadi E., Li J., Pang X., He J., Chang Y.X. (2021). Traditional herbal medicine: Therapeutic potential in rheumatoid arthritis. J. Ethnopharmacol..

[13] Behl T., Mehta K., Sehgal A., Singh S., Sharma N., Ahmadi A., Arora S., Bungau S. (2021). Exploring the role of polyphenols in rheumatoid arthritis. Crit. Rev. Food Sci. Nutr..

[14] Harari S., Spagnolo P., Cocconcelli E., Luisi F., Cottin V. (2018). Recent advances in the pathobiology and clinical management of lymphangioleiomyomatosis. Curr. Opin. Pulm. Med..

[15] Weill D., Benden C., Corris P.A., Dark J.H., Davis R.D., Keshavjee S., Lederer D.J., Mulligan M.J., Patterson G.A., Singer L.G. (2015). A consensus document for the selection of lung transplant candidates: 2014—An update from the Pulmonary Transplantation Council of the International Society for Heart and Lung Transplantation. J. Heart Lung Transpl..

[16] Aletaha D., Neogi T., Silman A.J., Funovits J., Felson D.T., Bingham C.O., Birnbaum N.S., Burmester G.R., Bykerk V.P., Cohen M.D. (2010). 2010 Rheumatoid arthritis classification criteria: An American College of Rheumatology/European League Against Rheumatism collaborative initiative. Arthritis Rheum..

[17] Smolen J.S., Landewé R.B.M., Bijlsma J.W.J., Burmester G.R., Dougados M., Kerschbaumer A., McInnes I.B., Sepriano A., van Vollenhoven R.F., de Wit M. (2020). EULAR recommendations for the management of rheumatoid arthritis with synthetic and biological disease-modifying antirheumatic drugs: 2019 update. Ann. Rheum. Dis..

[18] Radner H., Neogi T., Smolen J.S., Aletaha D. (2014). Performance of the 2010 ACR/EULAR classification criteria for rheumatoid arthritis: A systematic literature review. Ann. Rheum. Dis..

[19] Nannini C., Ryu J.H., Matteson E.L. (2008). Lung disease in rheumatoid arthritis. Curr. Opin. Rheumatol..

[20] Yamakawa H., Ogura T., Kameda H., Kishaba T., Iwasawa T., Takemura T., Kuwano K. (2021). Decision-Making Strategy for the Treatment of Rheumatoid Arthritis-Associated Interstitial Lung Disease (RA-ILD). J. Clin. Med..

[21] Lamattina A.M., Taveira-Dasilva A., Goldberg H.J., Bagwe S., Cui Y., Rosas I.O., Moss J., Henske E.P., El-Chemaly S. (2018). Circulating Biomarkers From the Phase 1 Trial of Sirolimus and Autophagy Inhibition for Patients With Lymphangioleiomyomatosis. Chest.

[22] Taillé C., Borie R., Crestani B. (2011). Current management of lymphangioleiomyomatosis. Curr. Opin. Pulm. Med..

[23] National Heart, Lung, and Blood Institute Lymphangioleiomyomatosis-Symptoms. https://www.nhlbi.nih.gov/health/lam/symptoms.

[24] O’Mahony A.M., Lynn E., Murphy D.J., Fabre A., McCarthy C. (2020). Lymphangioleiomyomatosis: A clinical review. Breathe.

[25] Gupta N., Lee H.S., Ryu J.H., Taveira-DaSilva A.M., Beck G.J., Lee J.C., McCarthy K., Finlay G.A., Brown K.K., Ruoss S.J. (2019). The NHLBI LAM Registry: Prognostic Physiologic and Radiologic Biomarkers Emerge From a 15-Year Prospective Longitudinal Analysis. Chest.

[26] Johnson S.R., Cordier J.F., Lazor R., Cottin V., Costabel U., Harari S., Reynaud-Gaubert M., Boehler A., Brauner M., Popper H. (2010). European Respiratory Society guidelines for the diagnosis and management of lymphangioleiomyomatosis. Eur. Respir. J..

[27] Johnson S.R., Whale C.I., Hubbard R.B., Lewis S.A., Tattersfield A.E. (2004). Survival and disease progression in UK patients with lymphangioleiomyomatosis. Thorax.

[28] Brentani M.M., Carvalho C.R.R., Saldiva P.H., Pacheco M.M., Oshima C.T. (1984). Steroid receptors in pulmonary lymphangiomyomatosis. Chest.

[29] Juvet S.C., McCormack F.X., Kwiatkowski D.J., Downey G.P. (2006). Molecular pathogenesis of lymphangioleiomyomatosis: Lessons learned from orphans. Am. J. Respir. Cell Mol. Biol..

[30] Taveira-DaSilva A.M., Steagall W.K., Moss J. (2006). Lymphangioleiomyomatosis. Cancer Control.

[31] Lin Y.J., Anzaghe M., Schülke S. (2020). Update on the Pathomechanism, Diagnosis, and Treatment Options for Rheumatoid Arthritis. Cells.

[32] McCarthy C., Gupta N., Johnson S.R., Yu J.J., McCormack F.X. (2021). Lymphangioleiomyomatosis: Pathogenesis, clinical features, diagnosis, and management. Lancet Respir. Med..

[33] Scherer H.U., Häupl T., Burmester G.R. (2020). The etiology of rheumatoid arthritis. J. Autoimmun..

[34] Alivernini S., Firestein G.S., McInnes I.B. (2022). The pathogenesis of rheumatoid arthritis. Immunity.

[35] Lucas C., Perdriger A., Amé P. (2020). Definition of B cell helper T cells in rheumatoid arthritis and their behavior during treatment. Semin. Arthritis Rheum..

[36] McInnes I.B., Buckley C.D., Isaacs J.D. (2016). Cytokines in rheumatoid arthritis—shaping the immunological landscape. Nat. Rev. Rheumatol..

[37] Elemam N.M., Hannawi S., Maghazachi A.A. (2020). Role of Chemokines and Chemokine Receptors in Rheumatoid Arthritis. ImmunoTargets Ther..

[38] Geusens P. (2012). The role of RANK ligand/osteoprotegerin in rheumatoid arthritis. Ther. Adv. Musculoskelet. Dis..

[39] Carmona F.D., Martín J. (2018). The potential of PTPN22 as a therapeutic target for rheumatoid arthritis. Expert Opin. Ther. Targets.

[40] Elsby L.M., Orozco G., Denton J., Worthington J., Ray D.W., Donn R.P. (2010). Functional evaluation of TNFAIP3 (A20) in rheumatoid arthritis. Clin. Exp. Rheumatol..

[41] Holoshitz J. (2010). The rheumatoid arthritis HLA-DRB1 shared epitope. Curr. Opin. Rheumatol..

[42] Taveira-DaSilva A.M., Moss J. (2016). Epidemiology, pathogenesis and diagnosis of lymphangioleiomyomatosis. Expert Opin. Orphan Drugs.

[43] Darling T.N., Pacheco-Rodriguez G., Gorio A., Lesma E., Walker C., Moss J. (2010). Lymphangioleiomyomatosis and TSC2−/− cells. Lymphat. Res. Biol..

[44] Li Z.J., Ying X.J., Chen H.L., Ye P.J., Chen Z.L., Li G., Jiang H.F., Liu J., Zhou S.Z. (2013). Insulin-like growth factor-1 induces lymphangiogenesis and facilitates lymphatic metastasis in colorectal cancer. World J. Gastroenterol..

[45] Seyama K., Kumasaka T., Souma S., Sato T., Kurihara M., Mitani K., Tominaga S., Fukuchi Y. (2006). Vascular endothelial growth factor-D is increased in serum of patients with lymphangioleiomyomatosis. Lymphat. Res. Biol..

[46] Matsui K., Tatsuguchi A., Valencia J., Yu Z.X., Bechtle J., Beasley M.B., Avila N., Travis W.D., Moss J., Ferrans V.J. (2000). Extrapulmonary lymphangioleiomyomatosis (LAM): Clinicopathologic features in 22 cases. Hum. Pathol..

[47] Cobbold S.P., of Cellular Immunology P., William S. (2013). The mTOR pathway and integrating immune regulation. Immunology.

[48] Logginidou H., Ao X., Russo I., Henske E.P. (2000). Frequent estrogen and progesterone receptor immunoreactivity in renal angiomyolipomas from women with pulmonary lymphangioleiomyomatosis. Chest.

[49] Grzegorek I., Lenze D., Chabowski M., Janczak D., Szolkowska M., Langfort R., Szuba A., Dziegiel P. (2015). Immunohistochemical evaluation of pulmonary lymphangioleiomyomatosis. Anticancer Res..

[50] Combe B., Landewe R., Daien C.I., Hua C., Aletaha D., Álvaro-Gracia J.M., Bakkers M., Brodin N., Burmester G.R., Codreanu C. (2017). 2016 update of the EULAR recommendations for the management of early arthritis. Ann. Rheum. Dis..

[51] Aletaha D., Smolen J.S. (2018). Diagnosis and Management of Rheumatoid Arthritis: A Review. JAMA.

[52] Pincus T., Sokka T. (2009). Laboratory Tests to Assess Patients with Rheumatoid Arthritis: Advantages and Limitations. Rheum. Dis. Clin. N. Am..

[53] D’Agostino M.A., Haavardsholm E.A., van der Laken C.J. (2016). Diagnosis and management of rheumatoid arthritis; What is the current role of established and new imaging techniques in clinical practice?. Best Pract. Res. Clin. Rheumatol..

[54] Xu K.F., Lo B.H. (2014). Lymphangioleiomyomatosis: Differential diagnosis and optimal management. Ther. Clin. Risk Manag..

[55] Hayashi T., Kumasaka T., Mitani K., Okada Y., Kondo T., Date H., Chen F., Oto T., Miyoshi S., Shiraishi T. (2016). Bronchial involvement in advanced stage lymphangioleiomyomatosis: Histopathologic and molecular analyses. Hum. Pathol..

[56] Gupta N., Meraj R., Tanase D., James L.E., Seyama K., Lynch D.A., Akira M., Meyer C.A., Ruoss S.J., Burger C.D. (2015). Accuracy of chest high-resolution computed tomography in diagnosing diffuse cystic lung diseases. Eur. Respir. J..

[57] Hayashida M., Seyama K., Inoue Y., Fujimoto K., Kubo K. (2007). The epidemiology of lymphangioleiomyomatosis in Japan: A nationwide cross-sectional study of presenting features and prognostic factors. Respirology.

[58] Ando K., Kurihara M., Kataoka H., Ueyama M., Togo S., Sato T., Doi T., Iwakami S.I., Takahashi K., Seyama K. (2013). Efficacy and safety of low-dose sirolimus for treatment of lymphangioleiomyomatosis. Respir. Investig..

[59] McCormack F.X., Inoue Y., Moss J., Singer L.G., Strange C., Nakata K., Barker A.F., Chapman J.T., Brantly M.L., Stocks J.M. (2011). Efficacy and safety of sirolimus in lymphangioleiomyomatosis. N. Engl. J. Med..

[60] Rapamune^®^ (sirolimus) Orphan Medicinal Product for the Treatment of Sporadic Lymphangioleiomyomatosis. https://www.ema.europa.eu/en/medicines/human/orphan-designations/eu-3-16-1704.

[61] McCormack F.X., Gupta N., Finlay G.R., Young L.R., Taveira-Da Silva A.M., Glasgow C.G., Steagall W.K., Johnson S.R., Sahn S.A., Ryu J.H. (2016). Official American Thoracic Society/Japanese Respiratory Society Clinical Practice Guidelines: Lymphangioleiomyomatosis Diagnosis and Management. Am. J. Respir. Crit. Care Med..

[62] Huang J., Manning B.D. (2009). A complex interplay between Akt, TSC2 and the two mTOR complexes. Biochem. Soc. Trans..

[63] Oprescu N., McCormack F.X., Byrnes S., Kinder B.W. (2013). Clinical predictors of mortality and cause of death in lymphangioleiomyomatosis: A population-based registry. Lung.

[64] Sathirareuangchai S., Shimizu D., Vierkoetter K.R. (2020). Pulmonary Lymphangioleiomyomatosis: A Case Report and Literature Review. Hawai’i J. Health Soc. Welf..

[65] Yoon H.Y., Hwang J.J., Kim D.S., Song J.W. (2018). Efficacy and safety of low-dose Sirolimus in Lymphangioleiomyomatosis. Orphanet J. Rare Dis..

[66] Wang Q., Luo M., Xiang B., Chen S., Ji Y. (2020). The efficacy and safety of pharmacological treatments for lymphangioleiomyomatosis. Respir. Res..

[67] Criner R.N., Al-abcha A., Lambert A.A., Han M.L.K. (2021). Lung Diseases Unique to Women. Clin. Chest Med..

[68] Moses M.A., Harper J., Folkman J. (2006). Doxycycline treatment for lymphangioleiomyomatosis with urinary monitoring for MMPs. N. Engl. J. Med..

[69] Johnson J., Johnson S.R. (2019). Cross-sectional study of reversible airway obstruction in LAM: Better evidence is needed for bronchodilator and inhaled steroid use. Thorax.

[70] Krymskaya V.P., Courtwright A.M., Fleck V., Dorgan D., Kotloff R., McCormack F.X., Kreider M. (2020). A phase II clinical trial of the Safety Of Simvastatin (SOS) in patients with pulmonary lymphangioleiomyomatosis and with tuberous sclerosis complex. Respir. Med..

[71] El-Chemaly S., Taveira-DaSilva A., Bagwe S., Klonowska K., Machado T., Lamattina A.M., Goldberg H.J., Jones A.M., Julien-Williams P., Maurer R. (2020). Celecoxib in lymphangioleiomyomatosis: Results of a phase I clinical trial. Eur. Respir. J..

[72] Steagall W.K., Zhang L., Cai X., Pacheco-Rodriguez G., Moss J. (2015). Genetic heterogeneity of circulating cells from patients with lymphangioleiomyomatosis with and without lung transplantation. Am. J. Respir. Crit. Care Med..

[73] Committee on Hospital Care, Section on Surgery, and Section on Critical Care (2010). Policy statement—Pediatric organ donation and transplantation. Pediatrics.

[74] Park J.E., Kim S.Y., Song J.H., Kim Y.S., Chang J., Lee J.G., Paik H.C., Park M.S. (2018). Comparison of short-term outcomes for connective tissue disease-related interstitial lung disease and idiopathic pulmonary fibrosis after lung transplantation. J. Thorac. Dis..

[75] Khawar M.U., Yazdani D., Zhu Z., Jandarov R., Dilling D.F., Gupta N. (2019). Clinical outcomes and survival following lung transplantation in patients with lymphangioleiomyomatosis. J. Heart Lung Transplant..

[76] Oishi H., Watanabe T., Matsuda Y., Noda M., Ejima Y., Saiki Y., Seyama K., Kondo T., Okada Y. (2018). Single lung transplantation for lymphangioleiomyomatosis: A single-center experience in Japan. Surg. Today.

[77] Kpodonu J., Massad M.G., Chaer R.A., Caines A., Evans A., Snow N.J., Geha A.S. (2005). The US experience with lung transplantation for pulmonary lymphangioleiomyomatosis. J. Heart Lung Transplant..

[78] Sirbu E., Buleu F., Tudor A., Dragan S. (2020). Vitamin D and disease activity in rheumatoid arthritis patients: A retrospective study in a Romanian cohort. Acta Biochim. Pol..

[79] Organ Transplant Reporting and Waiting List in Romania. http://transplant.ro/wp-content/uploads/2022/03/Lista-asteptare-2021.pdf.

[80] Behl T., Upadhyay T., Singh S., Chigurupati S., Alsubayiel A.M., Mani V., Vargas-De-la-cruz C., Uivarosan D., Bustea C., Sava C. (2021). Polyphenols Targeting MAPK Mediated Oxidative Stress and Inflammation in Rheumatoid Arthritis. Molecules.

[81] Szekanecz Z., McInnes I.B., Schett G., Szamosi S., Benkő S., Szűcs G. (2021). Autoinflammation and autoimmunity across rheumatic and musculoskeletal diseases. Nat. Rev. Rheumatol..

[82] Antin-Ozerkis D., Evans J., Rubinowitz A., Homer R.J., Matthay R.A. (2010). Pulmonary manifestations of rheumatoid arthritis. Clin. Chest Med..

[83] Futami S., Arai T., Hirose M., Sugimoto C., Ikegami N., Akira M., Kasai T., Kitaichi M., Hayashi S., Inoue Y. (2018). Comorbid connective tissue diseases and autoantibodies in lymphangioleiomyomatosis: A retrospective cohort study. Orphanet J. Rare Dis..

[84] Shah K.V., Goyal S., Kumar S., Shah A.D., Rana Y. (2021). Pulmonary Lymphangioleiomyomatosis and Role of Pleurodesis: Rare Case Reports. J. Clin. Diagn. Res..

[85] Ryu J.H., Moss J., Beck G.J., Lee J.C., Brown K.K., Chapman J.T., Finlay G.A., Olson E.J., Ruoss S.J., Maurer J.R. (2006). The NHLBI lymphangioleiomyomatosis registry: Characteristics of 230 patients at enrollment. Am. J. Respir. Crit. Care Med..

[86] Taylor P.C. (2002). VEGF and imaging of vessels in rheumatoid arthritis. Arthritis Res..

[87] Gupta N., Finlay G.A., Kotloff R.M., Strange C., Wilson K.C., Young L.R., Taveira-DaSilva A.M., Johnson S.R., Cottin V., Sahn S.A. (2017). Lymphangioleiomyomatosis Diagnosis and Management: High-Resolution Chest Computed Tomography, Transbronchial Lung Biopsy, and Pleural Disease Management. An Official American Thoracic Society/Japanese Respiratory Society Clinical Practice Guideline. Am. J. Respir. Crit. Care Med..

[88] Gerosa M., De Angelis V., Riboldi P., Meroni P.L. (2008). Rheumatoid arthritis: A female challenge. Women’s Health.

[89] Prizant H., Taya M., Lerman I., Light A., Sen A., Mitra S., Foster T.H., Hammes S.R. (2016). Estrogen maintains myometrial tumors in a lymphangioleiomyomatosis model. Endocr. Relat. Cancer.

[90] Itoh Y. (2017). Metalloproteinases in Rheumatoid Arthritis: Potential Therapeutic Targets to Improve Current Therapies. Prog. Mol. Biol. Transl. Sci..

[91] Ferrans V.J., Yu Z.X., Nelson W.K., Valencia J.C., Tatsuguchi A., Avila N.A., Riemenschn W., Matsui K., Travis W.D., Moss J. (2000). Lymphangioleiomyomatosis (LAM): A review of clinical and morphological features. J. Nippon Med. Sch..

[92] Laragione T., Gulko P.S. (2010). mTOR regulates the invasive properties of synovial fibroblasts in rheumatoid arthritis. Mol. Med..

[93] Park S.Y., Lee S.W., Kim H.Y., Lee W.S., Hong K.W., Kim C.D. (2015). HMGB1 induces angiogenesis in rheumatoid arthritis via HIF-1α activation. Eur. J. Immunol..

[94] Dodd K.M., Yang J., Shen M.H., Sampson J.R., Tee A.R. (2015). mTORC1 drives HIF-1α and VEGF-A signalling via multiple mechanisms involving 4E-BP1, S6K1 and STAT3. Oncogene.

